# Myeloid-Adaptive Response to Atezolizumab (MARTA): A Two-Gene CXCL13–CXCL8 Transcriptomic Biomarker Across Renal Cell and Urothelial Carcinoma

**DOI:** 10.3390/cimb48101012

**Published:** 2026-10-01

**Authors:** Mario Uccello

**Affiliations:** Royal United Hospitals Bath NHS Foundation Trust, Bath BA1 3NG, UK; mario_uccello@hotmail.it

**Keywords:** atezolizumab, immune-checkpoint blockade, transcriptomic biomarker, CXCL13, CXCL8

## Abstract

Transcriptomic response biomarkers for immune-checkpoint blockade remain poorly defined across cancers. We developed the two-gene Myeloid-Adaptive Response to Atezolizumab (MARTA) score, integrating CXCL13 and CXCL8 expression. MARTA was developed in 81 atezolizumab-treated patients in IMmotion150, evaluated across treatment groups, and independently assessed in IMvigor210. In IMmotion150, higher MARTA was associated with higher objective response rate (ORR) with atezolizumab alone (odds ratio [OR], 3.20; 95% confidence interval [CI], 1.42–7.19) and atezolizumab plus bevacizumab (OR, 2.61; 95% CI, 1.43–4.76), but not with sunitinib. Higher MARTA was associated with longer progression-free survival (PFS) in both atezolizumab-containing groups (hazard ratio [HR], 0.60 and 0.65), but not with sunitinib (interaction *p* = 0.01). In IMvigor210, higher MARTA was associated with higher ORR (OR per 1-standard-deviation increase, 1.38; 95% CI, 1.05–1.81) and longer overall survival (OS) (HR, 0.76; 95% CI, 0.67–0.86). The OS association remained after multivariate adjustment (HR, 0.81; 95% CI, 0.69–0.95). Discrimination for ORR and OS, assessed by the area under the curve (AUC) and C-index, respectively, was modest for MARTA, tGE8, and the IFN-γ signature. These findings support further evaluation of MARTA, while prospective validation and cross-platform analytical reproducibility remain necessary before clinical application.

## 1. Introduction

Immune-checkpoint inhibitors have substantially improved the treatment of several advanced malignancies, but clinical benefit varies considerably among patients, and robust predictive biomarkers remain limited [1,2]. Programmed death ligand 1 (PD-L1) expression is the most widely used tissue biomarker in this setting, although its predictive accuracy is modest and highly context-dependent [3]. Tumour mutational burden (TMB) has also been associated with response to checkpoint inhibition, but its clinical performance is influenced by tumour type, assay methodology, and the threshold used for its definition [4]. These limitations support the need for biomarkers that more directly reflect tumour biology and the immune microenvironment. Gene-expression profiling allows immune-related and tumour-associated transcriptional signatures to be assessed within the same specimen [5]. This approach may be particularly informative in renal cell and urothelial carcinomas, where response to immune-checkpoint blockade appears to reflect distinct features of both the tumour and its immune microenvironment. In renal cell carcinoma, transcriptomic analyses from the IMmotion trials identified distinct angiogenic, immune, and myeloid-associated molecular profiles associated with differential outcomes following immune-checkpoint and VEGF-targeted therapy [6,7]. In metastatic urothelial carcinoma, analysis of IMvigor210 showed that response to atezolizumab was associated with a CD8 T-effector phenotype, whereas stromal TGF-β signalling was linked to T-cell exclusion and treatment resistance [8]. Together, these findings support the development of composite transcriptional biomarkers that integrate antitumour immune activity with intrinsic tumour biology and the microenvironment. Among transcriptomic signatures evaluated in atezolizumab studies, the eight-gene T-effector gene-expression signature (tGE8) is one of the best characterised and reflects IFN-γ signalling and CD8+ effector T-cell activity [8,9]. Its association with response has subsequently been examined across tumour types, although its predictive performance has not been consistent [10]. T-cell-inflamed tumours are not uniformly sensitive to checkpoint inhibition, suggesting that T-effector signatures may not fully capture the biological heterogeneity associated with treatment outcome [10,11,12]. Moreover, relatively parsimonious transcriptional signatures have shown reproducible associations with checkpoint-inhibitor outcomes across multiple tumour types [11,13]. In this study, we developed MARTA, the Myeloid-Adaptive Response to Atezolizumab score, a two-gene transcriptomic score based on the relative expression of CXCL13 and CXCL8. MARTA was derived in the randomised IMmotion150 renal cell carcinoma dataset, where its added value beyond tGE8 was assessed. We subsequently evaluated MARTA in the independent IMvigor210 cohort of patients with locally advanced or metastatic urothelial carcinoma treated with atezolizumab. Our aim was to determine whether a simple transcriptional score could identify a reproducible signal associated with outcome during PD-L1 blockade across different clinical endpoints and tumour types.

## 2. Materials and Methods

### 2.1. Analysis Cohorts and Clinical Endpoints

We conducted a retrospective biomarker study using de-identified clinical and tumour transcriptomic data from IMmotion150 and IMvigor210. IMmotion150 was used for exploratory biomarker development and evaluation across treatment groups, whereas IMvigor210 served as an independent cross-tumour evaluation cohort. IMmotion150 [6] was a randomised phase 2 study in previously untreated metastatic renal cell carcinoma, with patients assigned to sunitinib, atezolizumab monotherapy, or atezolizumab plus bevacizumab. IMvigor210 [14,15] was a phase 2 single-arm study of atezolizumab in patients with locally advanced or metastatic urothelial carcinoma. For the present analyses, patients with available pretreatment tumour transcriptomic data and the relevant clinical endpoints were included. Objective response rate (ORR) was derived from individual objective-response status recorded in the original trial data; for biomarker modelling, response status was analysed as a binary outcome. Progression-free survival (PFS) in IMmotion150 was measured from treatment assignment to disease progression or death, whichever occurred first. Overall survival (OS) in IMvigor210 was measured from treatment initiation to death from any cause. Patients without an event were censored at the last available follow-up.

### 2.2. MARTA Score Development

Gene-expression data were harmonised at the gene-symbol level. In IMmotion150, the publicly available transcripts-per-million (TPM) expression matrix from 263 molecularly evaluable tumours was transformed as log2(TPM + 1). The previously described tGE8 signature (IFNG, CXCL9, CD8A, GZMA, GZMB, CXCL10, PRF1, and TBX21) and the six-gene IFN-γ signature (IDO1, CXCL10, CXCL9, HLA-DRA, STAT1, and IFNG) [11] were reconstructed as immune-expression comparators. Within each cohort, expression of each constituent gene was standardised across all samples included in the molecular analysis using the cohort-specific mean and population standard deviation (SD). Each signature was calculated as the unweighted arithmetic mean of its gene-specific z scores. Standardisation was applied to log2(TPM + 1) values in IMmotion150 and to the normalised log2-expression values generated by mySimpleVoom in IMvigor210. A separate literature-informed exploratory panel of 44 genes with reported associations with sensitivity or resistance to immune-checkpoint blockade was assembled for candidate-gene selection, excluding genes included in tGE8 to avoid overlap. Selection of candidate genes was restricted to IMmotion150 patients receiving atezolizumab monotherapy who were evaluable for ORR. Candidate genes were evaluated in two sequential steps. At each step, genes were added separately to a logistic regression model containing continuous tGE8 and any gene retained at the preceding step, and were ranked according to the incremental likelihood-ratio statistic (Table S1). The procedure was exploratory, and no multiplicity-adjusted significance threshold was applied. MARTA was constructed using equal absolute weights for CXCL13 and CXCL8 rather than outcome-derived gene-specific coefficients. For each tumour, CXCL13 and CXCL8 expression was transformed as log2(TPM + 1) and standardised separately using the mean and population SD of the IMmotion150 molecular cohort. MARTA was defined as the standardised difference between the gene-specific z scores:

*MARTA* = *z*[*z*(*log*2(*TPM*_*CXCL*13 + 1)) − *z*(*log*2(*TPM*_*CXCL*8 + 1))],

where z(X) denotes standardisation using the cohort mean and population SD. For exact reproduction of the IMmotion150 implementation, the fixed reference values were mean CXCL13 = 2.854063, SD CXCL13 = 0.6234777, mean CXCL8 = 3.001138, SD CXCL8 = 0.4441556, and SD of the resulting CXCL13-minus-CXCL8 z-score difference = 1.240428; the mean of this difference was effectively zero. Accordingly, MARTA was calculated directly as:

*MARTA* = {[(*log*2(*TPM*_*CXCL*13 + 1) − 2.854063)/0.6234777] − [(*log*2(*TPM*_*CXCL*8 + 1) − 3.001138)/0.4441556]}/1.240428.

Higher MARTA values therefore reflected greater CXCL13 expression relative to CXCL8 after standardisation. To assess robustness to the normalisation strategy, an alternative unstandardised CXCL13/CXCL8 log-ratio was calculated as log2(TPM_CXCL13 + 1) − log2(TPM_CXCL8 + 1), without gene-specific or score standardisation. For categorical analyses, the upper tertile of MARTA in the complete IMmotion150 molecular cohort was used as the fixed cutoff (0.4556475), with MARTA-high defined as >0.4556475 and MARTA-low as ≤0.4556475. The corresponding upper-tertile cutoff for the unstandardised log-ratio was 0.129762. tGE8 was not incorporated into the MARTA score and was used only as a comparator and adjustment covariate in secondary analyses.

### 2.3. Evaluation of MARTA in IMmotion150

Associations of MARTA with ORR and PFS in IMmotion150 were evaluated using logistic regression and Cox proportional-hazards regression, respectively. Analyses were performed separately within each treatment group, with MARTA evaluated primarily as a continuous variable and effect estimates reported per 1-SD increase in the score. Categorical analyses comparing MARTA-high with MARTA-low were also performed, with MARTA-low as the reference group. PFS distributions according to MARTA group were estimated using the Kaplan–Meier method. To assess whether the associations between MARTA and ORR or PFS differed among treatment groups, interaction models included continuous MARTA, treatment group, and their interaction. The overall interaction was assessed by likelihood-ratio comparison with the corresponding model without the interaction. To determine whether the associations of MARTA were maintained after accounting for tGE8, additional analyses in the atezolizumab-monotherapy group included continuous MARTA and continuous tGE8 in the same model.

### 2.4. Independent Evaluation of MARTA in IMvigor210

MARTA was calculated in IMvigor210 using the same equally weighted z[z(CXCL13) − z(CXCL8)] formulation after cohort-specific normalisation. For the normalisation-sensitivity analysis, the unstandardised CXCL13/CXCL8 log-ratio defined in Section 2.2 was calculated directly from the normalised expression values, without gene-specific or score standardisation. One transcriptomic sample per patient was retained before normalisation. RNA-sequencing counts were normalised using the DESeq-based implementation of mySimpleVoom in the IMvigor210CoreBiologies package. For the single patient with two available transcriptomic samples, the first sample in source-data order was used for the primary analysis; use of the alternative sample produced virtually identical results. Continuous MARTA was the primary evaluation variable. Associations of continuous MARTA with ORR and OS were evaluated using logistic regression and Cox proportional-hazards regression, respectively. Additional models for each endpoint included baseline Eastern Cooperative Oncology Group (ECOG) performance status, dichotomised as 0 versus >0. The continuous log-ratio was evaluated using the same models. For categorical analyses, the upper tertile of MARTA in the full molecular cohort was used as a fixed threshold (0.3871498) and applied unchanged to the population evaluable for ORR. In additional sensitivity analyses, the IMmotion150-derived cutoffs for MARTA (0.4556475) and the unstandardised log-ratio (0.129762) were applied unchanged to IMvigor210, and their associations with ORR and OS were reassessed. OS distributions according to MARTA group were estimated using the Kaplan–Meier method. In secondary multivariate analyses, continuous MARTA and the unstandardised log-ratio were each evaluated in separate models after adjustment for continuous tGE8, TMB, PD-L1 immune-cell expression, and ECOG performance status. Analyses were restricted to patients with complete data for all included covariates. Prior platinum exposure was examined in sensitivity analyses by including it as an additional covariate, evaluating the association between MARTA and OS within subgroups defined by prior platinum exposure, and testing the interaction between MARTA and prior platinum exposure. This interaction was also evaluated in the corresponding multivariate Cox model adjusted for tGE8, TMB, PD-L1 immune-cell expression, and ECOG performance status.

### 2.5. Statistical Analysis

Missing data were not imputed, and each analysis included only patients with available data for the variables required by the corresponding model. Effect estimates are reported as odds ratios (ORs) or hazard ratios (HRs), as appropriate, with 95% confidence intervals (CIs). The log-ratio was rescaled to 1 SD only for regression effect estimates. Discrimination of MARTA, the unstandardised CXCL13/CXCL8 log-ratio, and the comparator biomarkers CXCL13, CXCL8, tGE8, and the six-gene IFN-γ signature, each analysed as a continuous variable, was assessed using the area under the receiver operating characteristic curve (AUC) for ORR and Harrell’s concordance index (C-index) for time-to-event outcomes. AUC 95% CIs were calculated using the DeLong method, and C-index 95% CIs using a normal approximation based on the estimated standard error. For ORR analyses, higher CXCL8 expression was prespecified as indicating a lower probability of response, whereas higher values of the other biomarkers indicated a higher probability of response. The incremental value of MARTA over tGE8 was evaluated by comparing models containing tGE8 alone with models containing both tGE8 and MARTA and by comparing their AUC or C-index values. These analyses were conducted separately within each treatment group in IMmotion150 and in the overall IMvigor210 cohort. Spearman rank correlations among MARTA, CXCL13, CXCL8, tGE8, and the IFN-γ signature were calculated separately in the complete molecular cohorts. To assess potential compositional confounding, tumour purity was estimated from transcriptomic data using the Estimation of Stromal and Immune Cells in Malignant Tumour Tissues Using Expression Data (ESTIMATE) algorithm [16], and the ORR and survival models were repeated with continuous tumour-purity estimates as an additional covariate. Kaplan–Meier methods were used to estimate PFS and OS distributions and median survival. The proportional-hazards assumption for Cox models was assessed using scaled Schoenfeld residuals. In the atezolizumab-plus-bevacizumab PFS analysis, where evidence of non-proportional hazards was observed, an exploratory model including an interaction between continuous MARTA and log(time) was fitted to assess whether the association between MARTA and PFS varied over follow-up. In the atezolizumab-monotherapy PFS analysis, departure from linearity was assessed by comparing a model containing MARTA as a linear continuous variable with a model incorporating MARTA as a natural cubic spline with 3 degrees of freedom. Likelihood-ratio tests were used for nested-model comparisons, including treatment-interaction testing and evaluation of the incremental value of MARTA over tGE8. All *p* values were two-sided. Categorical and time-varying analyses were exploratory. Statistical analyses were performed using MedCalc Statistical Software version 23.5.2 (MedCalc Software Ltd., Ostend, Belgium) and R version 4.4.2 (R Foundation for Statistical Computing, Vienna, Austria) in RStudio version 2026.04.0+526 (Posit Software, PBC, Boston, MA, USA). ChatGPT (GPT-5.6 Sol; OpenAI, San Francisco, CA, USA) was used to assist with the formulation, refinement, and troubleshooting of complex R commands; all code execution, statistical outputs, and interpretation were reviewed and verified by the author.

## 3. Results

### 3.1. Analysis Cohorts, Clinical Endpoints and MARTA Development

In IMmotion150, 247 patients were evaluable for ORR and 263 for PFS. Of the patients evaluable for ORR, 81 received atezolizumab monotherapy and constituted the development population in which the 44 candidate genes were evaluated. In IMvigor210, 347 unique patients comprised the OS analysis population, of whom 298 were evaluable for ORR. Among the 44 candidate genes, CXCL13 ranked first at the initial step and CXCL8 ranked first after inclusion of CXCL13 (Table S1); these genes were retained for construction of MARTA. Higher CXCL13 expression was associated with higher ORR, whereas higher CXCL8 expression was associated with lower ORR. These opposing associations provided the basis for the equally weighted CXCL13-minus-CXCL8 formulation of the final score.

### 3.2. Evaluation of MARTA in IMmotion150

For ORR, higher continuous MARTA was associated with greater odds of objective response in the atezolizumab-monotherapy group (OR, 3.20; 95% CI, 1.42–7.19; *p* = 0.005) and the atezolizumab-plus-bevacizumab group (OR, 2.61; 95% CI, 1.43–4.76; *p* = 0.002), but not in the sunitinib group (OR, 1.16; 95% CI, 0.69–1.95; *p* = 0.58). The association between MARTA and ORR differed across treatment groups (overall interaction *p* = 0.05). Categorical analyses showed a concordant pattern (Table 1, Panel A). Higher continuous MARTA was also associated with longer PFS in the atezolizumab-monotherapy group (HR, 0.60; 95% CI, 0.48–0.75; *p* < 0.001) and the atezolizumab-plus-bevacizumab group (HR, 0.65; 95% CI, 0.48–0.89; *p* = 0.007), but not in the sunitinib group (HR, 1.01; 95% CI, 0.77–1.31; *p* = 0.96). The association between MARTA and PFS differed significantly across treatment groups (likelihood-ratio χ^2^ = 9.08; 2 df; *p* = 0.01) (Table 1, Panel B). Among patients receiving atezolizumab monotherapy, median PFS was 17.31 months in the MARTA-high group (95% CI, 5.45–not reached [NR]) and 5.35 months in the MARTA-low group (95% CI, 2.86–6.14), with an HR of 0.39 (95% CI, 0.21–0.71; *p* = 0.002) (Figure 1). The corresponding categorical HRs were 0.89 (95% CI, 0.49–1.61; *p* = 0.69) with sunitinib and 0.59 (95% CI, 0.34–1.04; *p* = 0.07) with atezolizumab plus bevacizumab. There was no evidence of violation of the proportional-hazards assumption for continuous MARTA in the atezolizumab-monotherapy or sunitinib groups. In the atezolizumab-plus-bevacizumab group, the MARTA-by-log(time) interaction was significant (*p* = 0.01), indicating that the favourable association between MARTA and PFS attenuated over follow-up. There was no evidence of departure from linearity in the atezolizumab-monotherapy group (*p* = 0.32). After adjustment for continuous tGE8 in the atezolizumab-monotherapy group, MARTA remained associated with objective response (adjusted OR, 3.97; 95% CI, 1.54–10.25; *p* = 0.004) and PFS (adjusted HR, 0.49; 95% CI, 0.36–0.66; *p* < 0.001), while the alternative unstandardised CXCL13/CXCL8 formulation showed concordant associations with ORR (adjusted OR, 6.39; 95% CI, 1.95–20.94; *p* = 0.002) and PFS (adjusted HR, 0.49; 95% CI, 0.36–0.68; *p* < 0.001) (Table S2). For ORR, the AUC was 0.743 (95% CI, 0.613–0.872) with atezolizumab monotherapy, 0.727 (95% CI, 0.606–0.848) with atezolizumab plus bevacizumab, and 0.535 (95% CI, 0.388–0.682) with sunitinib. For PFS, the corresponding C-index values were 0.668 (95% CI, 0.591–0.745), 0.628 (95% CI, 0.548–0.707), and 0.483 (95% CI, 0.400–0.565), respectively. The alternative CXCL13/CXCL8 formulation showed similar discrimination across the three treatment groups (Table S3). In the atezolizumab-monotherapy group, MARTA showed numerically higher ORR AUC and PFS C-index values than CXCL13, CXCL8, tGE8, and the IFN-γ signature. In the atezolizumab-plus-bevacizumab group, tGE8 and the IFN-γ signature showed slightly higher ORR AUCs than MARTA, whereas their PFS C-indices were similar. Adding MARTA to tGE8 increased the ORR AUC from 0.568 to 0.743 with atezolizumab monotherapy (likelihood-ratio *p* < 0.001) and from 0.749 to 0.779 with atezolizumab plus bevacizumab (*p* = 0.046), but produced little change with sunitinib (0.522 to 0.540; *p* = 0.62). For PFS, adding MARTA increased the C-index from 0.543 to 0.665 with atezolizumab monotherapy (*p* < 0.001), compared with smaller changes in the atezolizumab-plus-bevacizumab group (0.626 to 0.649; *p* = 0.094) and the sunitinib group (0.538 to 0.542; *p* = 0.94). In IMmotion150, MARTA was positively correlated with CXCL13 (ρ = 0.496), tGE8 (ρ = 0.424), and the IFN-γ signature (ρ = 0.382), and inversely correlated with CXCL8 (ρ = −0.661). tGE8 and the IFN-γ signature were strongly correlated (ρ = 0.928) (Table S4).

### 3.3. Independent Evaluation of MARTA in IMvigor210

In the 298 patients evaluable for ORR, higher continuous MARTA was associated with higher ORR (OR per 1-SD increase, 1.38; 95% CI, 1.05–1.81; *p* = 0.02). In categorical analyses, ORR values were 30.7% in the MARTA-high group and 18.8% in the MARTA-low group (OR, 1.92; 95% CI, 1.10–3.33; *p* = 0.02) (Table 2). Among the 347 IMvigor210 patients with available pretreatment transcriptomic data, higher continuous MARTA was associated with longer OS (HR per 1-SD increase, 0.76; 95% CI, 0.67–0.86; *p* < 0.001). The associations of continuous MARTA with ORR and OS both remained significant after adjustment for ECOG performance status (Table S2). Median OS values were 15.61 months in the MARTA-high group (95% CI, 10.41–NR) and 7.06 months in the MARTA-low group (95% CI, 6.24–9.00) (Figure 2), with an HR for death of 0.57 (95% CI, 0.43–0.77; *p* < 0.001) (Table 2). In a multivariate Cox analysis restricted to the 271 patients with available data for MARTA, tGE8, TMB, PD-L1 immune-cell expression, and ECOG performance status, among whom 172 deaths occurred, MARTA remained associated with OS after adjustment for these variables (adjusted HR, 0.81; 95% CI, 0.69–0.95; *p* = 0.01). The alternative non-z-standardised CXCL13/CXCL8 formulation was also associated with ORR (OR per 1-SD increase, 1.39; 95% CI, 1.06–1.82; *p* = 0.02) and OS (HR, 0.76; 95% CI, 0.67–0.86; *p* < 0.001). It remained associated with OS after multivariate adjustment (Table S2). Using the IMmotion150-derived cutoffs in IMvigor210, MARTA-high was associated with ORR (OR, 1.86; 95% CI, 1.06–3.26; *p* = 0.03) and OS (HR, 0.59; 95% CI, 0.43–0.79; *p* < 0.001). When the fixed IMmotion150-derived CXCL13/CXCL8 cutoff was applied, the corresponding estimates were OR 1.70 (95% CI, 0.97–2.97; *p* = 0.06) for ORR and HR 0.61 (95% CI, 0.46–0.82; *p* = 0.001) for OS (Table S2). The proportional-hazards assumption was not significantly violated for continuous MARTA (*p* = 0.06) or for the multivariate model (global *p* = 0.24). Prior platinum exposure did not materially alter the association between MARTA and OS. After adjustment for prior platinum exposure, the MARTA HR was 0.77 (95% CI, 0.67–0.87; *p* < 0.001), with no evidence that the association differed according to prior platinum exposure (interaction *p* = 0.71). The corresponding HRs were 0.76 (95% CI, 0.66–0.87) among patients with prior platinum exposure and 0.81 (95% CI, 0.58–1.12) among those without prior platinum exposure. In the multivariate model additionally adjusted for prior platinum exposure, the association between MARTA and OS was essentially unchanged, with no evidence of interaction with prior platinum exposure (*p* = 0.10) (Table S5). In discrimination analyses, the AUC and C-index were 0.598 and 0.572 for MARTA and 0.601 and 0.574 for the alternative CXCL13/CXCL8 formulation, respectively (Table S3). The corresponding values were 0.610 and 0.546 for tGE8 and 0.613 and 0.552 for the IFN-γ signature. Adding MARTA to tGE8 increased the ORR AUC from 0.610 to 0.626, without a significant improvement in model fit (likelihood-ratio *p* = 0.21), and increased the OS C-index from 0.546 to 0.572 (*p* = 0.001). MARTA was positively correlated with CXCL13 (ρ = 0.639), tGE8 (ρ = 0.442), and the IFN-γ signature (ρ = 0.406) and inversely correlated with CXCL8 (ρ = −0.647). The correlation between tGE8 and the IFN-γ signature was ρ = 0.957 (Table S4). Adjustment for ESTIMATE-derived tumour purity did not materially alter the observed associations of MARTA with ORR and PFS in IMmotion150 or with ORR and OS in IMvigor210 (Table S2, Panel C).

## 4. Discussion

In this exploratory biomarker study, MARTA, a two-gene score based on the relative expression of CXCL13 and CXCL8, was consistently associated with outcomes during atezolizumab treatment across two cohorts and two tumour types. In IMmotion150, higher continuous MARTA was associated with higher ORR with atezolizumab monotherapy and atezolizumab plus bevacizumab (ORs, 3.20 and 2.61, respectively) and with longer PFS in both atezolizumab-containing groups (HRs, 0.60 and 0.65, respectively), whereas no association with PFS was observed with sunitinib. The association between MARTA and outcome differed across treatment groups, with *p* = 0.05 for ORR and *p* = 0.01 for PFS. In the independent IMvigor210 cohort, MARTA was associated with both ORR and OS during atezolizumab treatment; both associations remained after adjustment for ECOG performance status, and the OS association also remained after adjustment for tGE8, TMB, PD-L1 expression, and ECOG performance status. These findings do not establish clinical utility, but their consistency across different tumour types and clinical endpoints, together with the treatment-group differences observed in IMmotion150, supports further investigation of MARTA as a potential marker of sensitivity to PD-L1 blockade. Discrimination was numerically higher in the atezolizumab-containing groups than with sunitinib in IMmotion150, consistent with the treatment-specific pattern observed in the regression analyses. However, performance was more limited in IMvigor210, with an AUC of 0.598 for ORR and a C-index of 0.572 for OS. These findings support an association with outcome but indicate that MARTA alone has limited discriminatory performance in an independent cohort. The positive component of MARTA, CXCL13, has substantial biological support as a marker of tumour-reactive adaptive immunity [17,18,19,20], with favourable clinical associations also reported across other tumour types [21,22]. Single-cell and tissue-based studies have linked CXCL13 to tumour-reactive CD8+ T cells and B-cell-rich tertiary lymphoid structures (TLS) associated with response to immune-checkpoint blockade across several cancers [17,23,24,25]. In advanced bladder cancer, higher CXCL13 expression was associated with ORR and longer survival during immune-checkpoint therapy, but not with outcome in patients who did not receive such treatment, and correlated with tumour TLS [26]. These findings provide disease-specific support for the favourable association of the CXCL13 component of MARTA observed in IMvigor210. High CXCL8 expression has been associated with adverse clinical features and outcomes and, in immunotherapy cohorts, with a myeloid-inflamed tumour microenvironment, increased neutrophil infiltration, and reduced clinical benefit [27,28,29,30,31]. In pooled analyses of atezolizumab trials and in an independent analysis of phase 3 clinical trials, high circulating IL-8 or tumour CXCL8 expression was associated with neutrophil-rich myeloid inflammation, reduced antigen-presentation machinery, and poorer outcomes [28,29]. Consistent with these findings, IMmotion150 showed different associations of T-effector/IFN-γ and myeloid inflammatory profiles with PFS across treatment groups, with myeloid inflammation proposed as a potential mechanism of resistance to checkpoint inhibition [6]. MARTA therefore integrates two biologically contrasting signals. CXCL13 is linked to tumour-reactive adaptive immunity and TLS, whereas CXCL8 is associated with myeloid inflammation and resistance to checkpoint inhibition. By using the standardised difference between CXCL13 and CXCL8, MARTA captures the balance between these signals rather than T-cell inflammation alone. This distinguishes MARTA from tGE8, which primarily reflects T-effector and IFN-γ-related activity. Head-to-head analyses showed that no biomarker performed consistently best across treatment groups. MARTA showed its strongest discrimination in the atezolizumab-monotherapy group, where adding it to tGE8 produced the clearest improvements in model fit and discrimination. The gains were smaller or absent in the other IMmotion150 groups and modest in IMvigor210. Together with the adjusted analyses, these results suggest that MARTA captures information partly complementary to tGE8, but do not demonstrate consistent superiority over established immune-expression signatures or sufficient incremental value for clinical use. This interpretation is consistent with previous analyses of IMmotion150 and IMvigor210, in which response to PD-L1 blockade was influenced not only by immune activation but also by myeloid, stromal, and tumour-intrinsic features [6,8,10]. The simplicity of MARTA should also be considered in relation to more complex transcriptomic predictors. A recently reported 49-gene LogitDA model achieved an AUC of 0.75 in the independent PCD4989g metastatic urothelial carcinoma dataset, compared with 0.65 for tGE8, 0.67 for an IFN-γ signature, and 0.70 for a T-cell-inflamed signature [32]. A direct comparison was not performed because the published gene list was incomplete; tGE8 was retained as the principal comparator because its composition and calculation could be reproduced consistently. More complex models may provide greater predictive accuracy but may also be more susceptible to overfitting. In a cross-tumour analysis, a weighted 58-gene model achieved an AUC of 0.99 in the training population but below 0.65 in an independent cohort [10]. These findings illustrate the difficulty of developing transferable transcriptomic predictors; the rationale for the two-gene formulation was parsimony and biological interpretability, rather than presumed superiority over larger signatures. MARTA was developed in renal-cell carcinoma and subsequently evaluated in urothelial carcinoma, making it less likely that the findings reflect only a tumour-specific transcriptional pattern. This is relevant given the substantial transcriptional heterogeneity reported across tumour types treated with the same checkpoint inhibitor [10,13]. In IMmotion150, MARTA was associated with PFS in both atezolizumab-containing groups but not with sunitinib, and the association between MARTA and PFS differed significantly across treatment groups. Although this does not establish predictive utility, it supports the possibility of a treatment-dependent association. IMvigor210 showed a concordant association during atezolizumab treatment, but its single-arm design cannot determine whether MARTA identifies greater benefit or a more favourable prognosis. Prior platinum exposure did not significantly modify the association with OS. The performance of transcriptomic biomarkers may potentially be improved by combining them with selected clinical variables. Transcriptomic data capture tumour and immune biology, whereas clinical characteristics provide complementary information on disease burden, systemic inflammation, metastatic distribution, and general condition [33,34,35,36,37,38,39]. In a retrospective study of 62 patients with metastatic urothelial carcinoma treated with PD-1 or PD-L1 inhibitors, low neutrophil-to-lymphocyte ratio, absence of visceral metastases, and a higher tumour single-nucleotide variant count were independently associated with clinical benefit. A model combining these three variables achieved a c-statistic of 0.90, although external validation was considered necessary [40]. These findings suggest that combining MARTA with a limited number of clinically relevant variables may possibly improve discrimination, rather than necessarily expanding the transcriptomic component of the score. Formal evaluation of such an approach will require datasets containing both pretreatment transcriptomics and sufficiently complete clinical information, which remain relatively uncommon in publicly accessible immunotherapy cohorts. Contemporary treatment of advanced urothelial and renal cell carcinomas increasingly combines immune-checkpoint blockade with other agents [41,42,43]. This reinforces the need for prospectively validated biomarkers capable of informing treatment selection or de-escalation [44,45]. This study has several limitations. MARTA was developed retrospectively, and candidate-gene selection was exploratory and performed in a relatively small atezolizumab-monotherapy population. No multiplicity-adjusted significance threshold was used for gene selection, and the cutoff used to define MARTA-high and MARTA-low requires independent validation. Results were similar using the alternative unstandardised CXCL13/CXCL8 formulation and the IMmotion150-derived fixed cutoffs, suggesting robustness to normalisation and threshold choice. However, reproducibility across sequencing methods, tissue-processing procedures, and expression platforms remains to be established. Adjustment for ESTIMATE-derived tumour purity did not materially alter the findings, although residual confounding by specific immune-cell populations cannot be excluded. All immunotherapy analyses involved atezolizumab, and the findings may not generalise to other PD-1 or PD-L1 inhibitors. In addition, IMvigor210 lacked a non-immunotherapy comparator, limiting its ability to establish treatment specificity; validation in an independent randomised study would provide stronger evidence of a predictive effect. Although MARTA was consistently associated with outcome, its modest AUC and C-index in IMvigor210 indicate limited discrimination when used as a standalone biomarker. Finally, more complex transcriptomic models may achieve greater discrimination [32], whereas incomplete clinical annotation limited adjustment for clinical variables in IMvigor210 to ECOG performance status and prior platinum exposure and precluded comparable adjustment in IMmotion150. The potential value of integrating transcriptomic and clinical information has been demonstrated in atezolizumab-treated urothelial carcinoma and warrants further evaluation in datasets with sufficiently complete molecular and clinical data [46].

## 5. Conclusions

MARTA, a simple two-gene transcriptomic score, was associated with clinical outcomes during atezolizumab treatment in renal cell and urothelial carcinoma cohorts. These findings support further validation but are not sufficient to recommend MARTA for clinical decision-making, particularly given the modest discrimination observed in IMvigor210. Future studies should establish its analytical reproducibility, validate it in additional cohorts treated with PD-1 or PD-L1 inhibitors, and determine whether selected clinical variables can improve its discrimination while preserving the simplicity of the score.

## Figures and Tables

**Figure 1:**
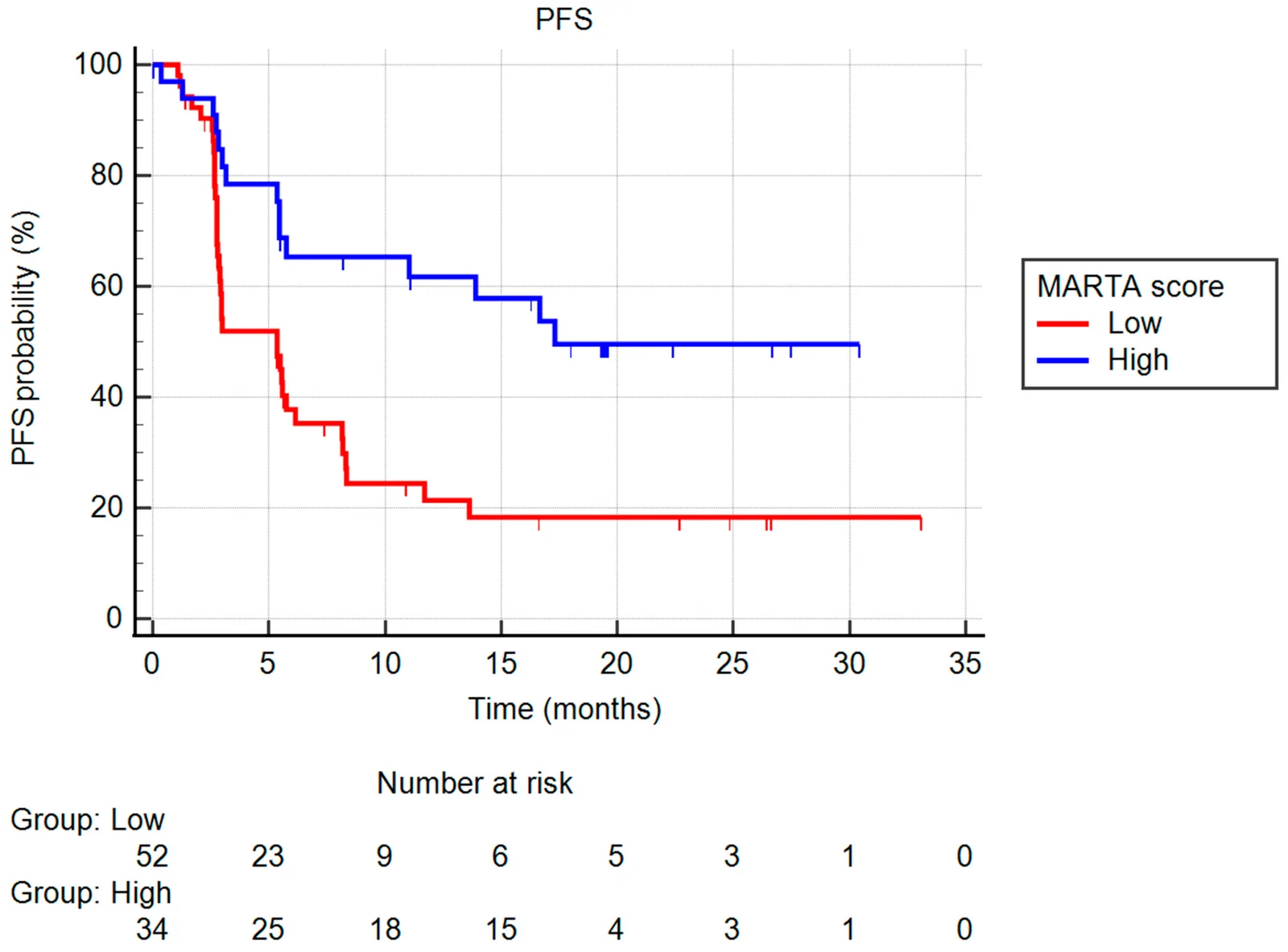
Progression-Free Survival According to MARTA Category in IMmotion150. Kaplan–Meier estimates of PFS among patients receiving atezolizumab monotherapy according to MARTA category. MARTA-high was defined as a score > 0.4556475 and MARTA-low as a score ≤ 0.4556475. Median PFS values were 17.31 months in the MARTA-high group (95% CI, 5.45–NR) and 5.35 months in the MARTA-low group (95% CI, 2.86–6.14). The HR for MARTA-high versus MARTA-low was 0.39 (95% CI, 0.21–0.71; *p* = 0.002). Tick marks indicate censored observations; numbers at risk are shown below the plot. Abbreviations: CI, confidence interval; HR, hazard ratio; MARTA, Myeloid-Adaptive Response to Atezolizumab; NR, not reached; PFS, progression-free survival.

**Figure 2:**
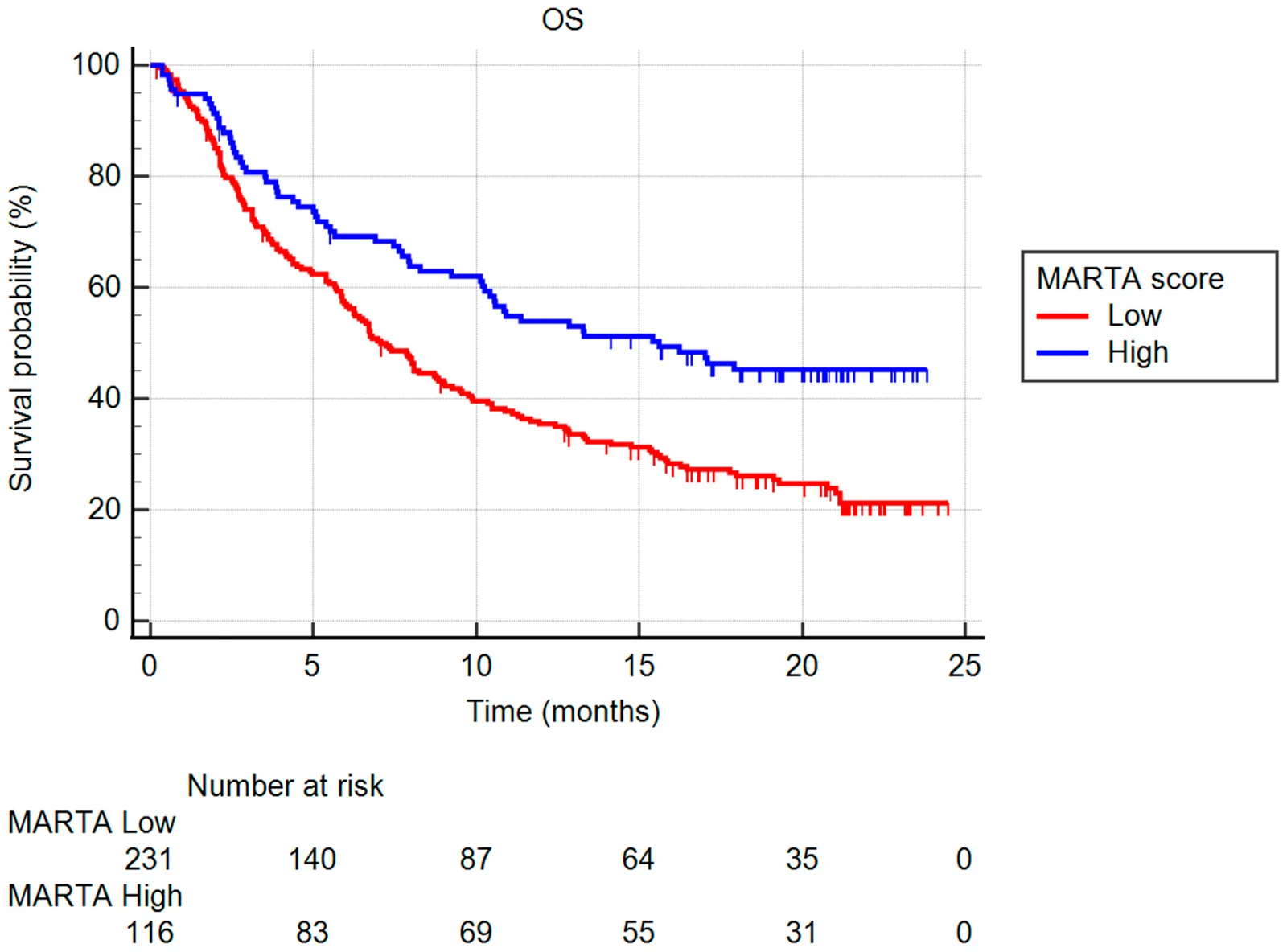
Overall Survival According to MARTA Category in IMvigor210. Kaplan–Meier estimates of OS according to MARTA category among 347 patients with available pretreatment transcriptomic data in IMvigor210. MARTA-high was defined as a MARTA score > 0.3871498 and MARTA-low as a score ≤ 0.3871498. Median OS values were 15.61 months in the MARTA-high group (95% CI, 10.41–NR) and 7.06 months in the MARTA-low group (95% CI, 6.24–9.00). The HR for death for MARTA-high versus MARTA-low was 0.57 (95% CI, 0.43–0.77; *p* < 0.001). Tick marks indicate censored observations; numbers at risk are shown below the plot. Abbreviations: CI, confidence interval; HR, hazard ratio; MARTA, Myeloid-Adaptive Response to Atezolizumab; NR, not reached; OS, overall survival.

**Table 1:** Association of MARTA with ORR and PFS in IMmotion150.

**Panel (A). ORR**							
**Treatment**		**ORR, n/N (%)**		**OR, MARTA-high vs. low** **(95% CI)**	***p* value**	**OR per 1-SD increase** **(95% CI)**	***p* value**
		**MARTA-low**	**MARTA-high**				
Sunitinib		15/60 (25.0)	9/22 (40.9)	2.08 (0.74–5.83)	0.17	1.16 (0.69–1.95)	0.58
Atezo		6/50 (12.0)	14/31 (45.2)	6.04 (1.99–18.29)	0.001	3.20 (1.42–7.19)	0.005
Atezo + Beva		11/54 (20.4)	17/30 (56.7)	5.11 (1.92–13.62)	0.001	2.61 (1.43–4.76)	0.002
**Panel (B). PFS**							
**Treatment**	**Events/N**	**Median PFS, months (95% CI)**		**HR, MARTA-high vs. low** **(95% CI)**	***p* value**	**HR per 1-SD increase** **(95% CI)**	***p* value**
		**MARTA-low**	**MARTA-high**				
Sunitinib	55/89	8.25 (4.43–10.81)	10.71 (3.71–22.34)	0.89 (0.49–1.61)	0.69	1.01 (0.77–1.31)	0.96
Atezo	51/86	5.35 (2.86–6.14)	17.31 (5.45–NR)	0.39 (0.21–0.71)	0.002	0.60 (0.48–0.75)	<0.001
Atezo + Beva	58/88	8.31 (2.92–11.40)	21.68 (9.86–25.07)	0.59 (0.34–1.04)	0.07	0.65 (0.48–0.89)	0.007

Continuous effect estimates are reported per 1-SD increase in MARTA; categorical ORs and HRs compare MARTA-high with MARTA-low. MARTA-high was defined by the fixed IMmotion150 upper-tertile cutoff (>0.4556475), and MARTA-low as ≤0.4556475. *p* values for interaction across treatment groups were 0.05 for ORR and 0.01 for PFS. In the Atezo + Beva PFS analysis, the proportional-hazards assumption was not satisfied; the continuous HR therefore represents an average association over follow-up. Abbreviations: Atezo, atezolizumab; Beva, bevacizumab; CI, confidence interval; HR, hazard ratio; MARTA, Myeloid-Adaptive Response to Atezolizumab; NR, not reached; OR, odds ratio; ORR, objective response rate; PFS, progression-free survival; SD, standard deviation.

**Table 2:** Independent evaluation of MARTA in IMvigor210.

MARTA Group	ORR, n/N (%)	OR for Response (95% CI); *p*	Deaths, n/N (%)	Median OS, Months (95% CI)	HR for Death (95% CI); *p*
MARTA-low	37/197 (18.8)	Reference	170/231 (73.6)	7.06 (6.24–9.00)	Reference
MARTA-high	31/101 (30.7)	1.92 (1.10–3.33); *p* = 0.02	61/116 (52.6)	15.61 (10.41–NR)	0.57 (0.43–0.77); *p* < 0.001

MARTA-high was defined using the upper-tertile cutoff from the full 347-patient IMvigor210 molecular cohort; the same threshold was applied unchanged to the 298-patient ORR population. In continuous analyses, each 1-SD increase in MARTA was associated with higher ORR (OR, 1.38; 95% CI, 1.05–1.81; *p* = 0.02) and longer OS (HR for death, 0.76; 95% CI, 0.67–0.86; *p* < 0.001). Abbreviations: CI, confidence interval; HR, hazard ratio; MARTA, Myeloid-Adaptive Response to Atezolizumab; NR, not reached; OR, odds ratio; ORR, objective response rate; OS, overall survival; SD, standard deviation.

## Data Availability

The clinical and tumour-transcriptomic data analysed in this study are publicly available through cBioPortal for Cancer Genomics [47,48,49] as iAtlas-harmonized datasets, under the study identifiers rcc_iatlas_immotion150_2018 and blca_iatlas_imvigor210_2017, corresponding to IMmotion150 and IMvigor210, respectively. IMvigor210 RNA-sequencing data were processed using the IMvigor210CoreBiologies R package, as described in the Methods. No new patient-level clinical or molecular data were generated in the present study.

## Supplementary Materials

The following supporting information can be downloaded at: https://www.mdpi.com/article/10.3390/cimb48101012/s1.
