# Supplementary material for: Myeloid-Adaptive Response to Atezolizumab (MARTA): A Two-Gene CXCL13–CXCL8 Transcriptomic Biomarker Across Renal Cell and Urothelial Carcinoma
Source: Curr Issues Mol Biol. 2026 Oct 1;48(10):1012. doi: 10.3390/cimb48101012 (PMC13703666; doi:10.3390/cimb48101012)
Supplement: Supplementary file 1 [file cimb-48-01012-s001.zip › cimb-4535799-supplementary.pdf]

Table S1. Candidate genes and sequential gene-selection results during MARTA development

**Domain**	**Gene**	**Step 1 rank**	**Step 1 LR χ^2 (P)**	**Step 2 rank**	**Step 2 LR χ^2 (P)**
Checkpoint/T-cell	CD274	19	1.25 (0.264)	35	0.25 (0.615)
	PDCD1	8	3.21 (0.073)	5	3.09 (0.079)
	CTLA4	28	0.60 (0.438)	41	0.00 (0.957)
	LAG3	20	0.97 (0.325)	11	1.49 (0.222)
	TIGIT	7	3.36 (0.067)	20	0.92 (0.339)
	HAVCR2	13	2.30 (0.129)	8	2.28 (0.131)
	ICOS	18	1.27 (0.260)	18	1.20 (0.274)
	CD27	2	3.87 (0.049)	17	1.20 (0.273)
	TNFRSF9	43	0.00 (0.977)	23	0.71 (0.400)
Adaptive/TLS/NK	**CXCL13**	1	6.38 (0.012)	—	—
	CCL5	17	1.35 (0.245)	14	1.34 (0.246)
	NKG7	3	3.84 (0.050)	4	3.18 (0.075)
	GNLY	27	0.61 (0.435)	36	0.25 (0.617)
Antigen/IFN	B2M	15	1.80 (0.180)	9	1.84 (0.175)
	HLA-A	21	0.88 (0.349)	15	1.34 (0.247)
	HLA-B	12	2.35 (0.125)	12	1.40 (0.236)
	HLA-C	6	3.44 (0.064)	6	3.07 (0.080)
	HLA-DRA	16	1.71 (0.192)	16	1.32 (0.251)
	TAP1	32	0.20 (0.652)	39	0.11 (0.744)
	TAP2	22	0.87 (0.352)	37	0.25 (0.620)
	JAK1	26	0.62 (0.430)	26	0.46 (0.500)
	JAK2	42	0.00 (0.975)	40	0.04 (0.836)
	STAT1	9	3.17 (0.075)	2	7.44 (0.006)
	IRF1	23	0.84 (0.360)	13	1.39 (0.239)
	NLRC5	29	0.31 (0.575)	28	0.43 (0.514)
Dendritic cell	BATF3	41	0.01 (0.938)	30	0.40 (0.528)
	XCR1	5	3.49 (0.062)	7	3.06 (0.080)
	CLEC9A	14	1.98 (0.159)	19	1.14 (0.286)
Myeloid/IS	TREM2	33	0.19 (0.662)	31	0.35 (0.554)
	SPP1	10	3.05 (0.081)	3	6.63 (0.010)
	APOE	24	0.70 (0.401)	34	0.27 (0.604)
	CSF1R	37	0.14 (0.711)	33	0.31 (0.578)
	LILRB1	35	0.18 (0.672)	42	0.00 (0.979)
	LILRB2	38	0.07 (0.790)	27	0.44 (0.509)
	FCGR3A	25	0.67 (0.414)	22	0.76 (0.382)
	**CXCL8**	4	3.70 (0.055)	1	9.39 (0.002)
	IL10	36	0.15 (0.703)	32	0.35 (0.555)
	TGFB1	31	0.21 (0.648)	24	0.53 (0.466)
Angiogenesis	VEGFA	34	0.18 (0.672)	43	0.00 (0.983)
	KDR	40	0.01 (0.919)	29	0.41 (0.523)
	FLT1	44	0.00 (0.980)	25	0.46 (0.498)
	ANGPT2	39	0.03 (0.860)	38	0.13 (0.722)
WNT/exclusion	WNT5A	30	0.31 (0.580)	21	0.86 (0.354)
	AXIN2	11	2.75 (0.097)	10	1.57 (0.210)

Candidate-gene selection was conducted in 81 patients receiving atezolizumab monotherapy who were evaluable for objective response. At step 1, each of the 44 candidate genes was added separately to a logistic regression model containing continuous tGE8 and ranked by the incremental likelihood-ratio statistic; CXCL13 ranked first and was retained. At step 2, each of the remaining 43 genes was added separately to the model containing tGE8 and CXCL13; CXCL8 ranked first and was retained. P values were not adjusted for multiplicity and should be interpreted as exploratory. A dash indicates that CXCL13 was retained at step 1 and was therefore not re-evaluated at step 2. **Abbreviations:** IFN, interferon; IS, immunosuppression; LR, likelihood ratio; NK, natural killer; ORR, objective response rate; tGE8, eight-gene T-effector gene-expression signature; TLS, tertiary lymphoid structure.

Table S2. Adjusted and robustness analyses of MARTA

Panel A. Adjusted continuous analyses

Formulation	Study and population	Endpoint	Adjustment covariates	Responses or events, n/N	Adjusted effect per 1-SD increase (95% CI)	P value
MARTA	IMmotion150, Atezo	ORR	tGE8	20/81	OR, 3.97 (1.54–10.25)	0.004
MARTA	IMmotion150, Atezo	PFS	tGE8	51/86	HR, 0.49 (0.36–0.66)	<0.001
MARTA	IMvigor210, Atezo	ORR	ECOG PS (>0 vs 0)	68/298	OR, 1.38 (1.05–1.81)	0.023
MARTA	IMvigor210, Atezo	OS	ECOG PS (>0 vs 0)	231/347	HR, 0.77 (0.68–0.87)	<0.001
MARTA	IMvigor210, Atezo	OS	tGE8, TMB, PD-L1, and ECOG PS	172/271	HR, 0.81 (0.69–0.95)	0.010
CXCL13/CXCL8 log-ratio	IMmotion150, Atezo	ORR	tGE8	20/81	OR, 6.39 (1.95–20.94)	0.002
CXCL13/CXCL8 log-ratio	IMmotion150, Atezo	PFS	tGE8	51/86	HR, 0.49 (0.36–0.68)	<0.001
CXCL13/CXCL8 log-ratio	IMvigor210, Atezo	OS	tGE8, TMB, PD-L1, and ECOG PS	172/271	HR, 0.80 (0.68–0.95)	0.009

Panel B. Fixed-threshold analyses in IMvigor210

Formulation	Fixed IMmotion150 cutoff	Endpoint	Low group	High group	Effect, high vs low (95% CI)	P value
MARTA	>0.4556475	ORR	40/207 (19.3%)	28/91 (30.8%)	OR, 1.86 (1.06–3.26)	0.031
MARTA	>0.4556475	OS	n=242; median 7.85 mo	n=105; median 16.23 mo	HR, 0.59 (0.43–0.79)	<0.001
CXCL13/CXCL8 log-ratio	>0.129762	ORR	39/199 (19.6%)	29/99 (29.3%)	OR, 1.70 (0.97–2.97)	0.062
CXCL13/CXCL8 log-ratio	>0.129762	OS	n=233; median 7.85 mo	n=114; median 15.61 mo	HR, 0.61 (0.46–0.82)	0.001

Panel C. Analyses adjusted for ESTIMATE-derived tumour purity

Study and population	ORR, responses n/N	Adjusted OR (95% CI); P value	Survival endpoint, events n/N	Adjusted HR (95% CI); P value
IMmotion150, Atezo	20/81	3.17 (1.41–7.15); 0.005	PFS, 51/86	0.58 (0.46–0.74); <0.001
IMmotion150, Atezo + Beva	28/84	2.55 (1.39–4.67); 0.002	PFS, 58/88	0.67 (0.48–0.92); 0.012
IMmotion150, Sunitinib	24/82	1.15 (0.67–1.95); 0.62	PFS, 55/89	1.01 (0.77–1.31); 0.96
IMvigor210, Atezo	68/298	1.54 (1.15–2.07); 0.004	OS, 231/347	0.74 (0.65–0.85); <0.001

Continuous effects are reported per 1-SD increase after adjustment for the listed covariates. The alternative non-z-standardised CXCL13/CXCL8 formulation was calculated as log2(TPM_CXCL13 + 1) − log2(TPM_CXCL8 + 1) in IMmotion150 and as the difference between mySimpleVoom-normalised log2 expression values in IMvigor210. Fixed cutoffs derived from all IMmotion150 patients with available molecular data were applied unchanged to IMvigor210, after cohort-specific normalisation of MARTA. For ORR, the numerator denotes responses; for PFS and OS, it denotes events. In Panel B, ORR cells show responses/n (%), and OS cells show group size and median OS. ECOG PS was dichotomised as 0 versus >0. Panel C reports models adjusted for continuous ESTIMATE-derived tumour purity; the survival endpoint was PFS in IMmotion150 and OS in IMvigor210. **Abbreviations:** Atezo, atezolizumab; CI, confidence interval; ECOG PS, Eastern Cooperative Oncology Group performance status; ESTIMATE, Estimation of STromal and Immune cells in MAlignant Tumour tissues using Expression data; HR, hazard ratio; MARTA, Myeloid–Adaptive Response to Atezolizumab; OR, odds ratio; ORR, objective response rate; OS, overall survival; PD-L1, programmed death ligand 1; PFS, progression-free survival; SD, standard deviation; tGE8, eight-gene T-effector gene-expression signature; TMB, tumour mutational burden.

Table S3. Comparative discrimination of MARTA and immune-expression biomarkers

Panel A. AUC for ORR in IMmotion150

Biomarker	Atezo	Atezo + Beva	Sunitinib
**MARTA**	0.743 (0.613–0.872)	0.727 (0.606–0.848)	0.535 (0.388–0.682)
CXCL13/CXCL8 log-ratio	0.750 (0.622–0.878)	0.740 (0.620–0.859)	0.521 (0.374–0.668)
CXCL13	0.625 (0.498–0.751)	0.733 (0.610–0.856)	0.423 (0.281–0.565)
CXCL8	0.623 (0.481–0.765)	0.598 (0.466–0.730)	0.617 (0.483–0.751)
tGE8	0.568 (0.422–0.714)	0.749 (0.637–0.862)	0.522 (0.390–0.654)
IFN-γ	0.493 (0.347–0.640)	0.746 (0.635–0.856)	0.540 (0.411–0.668)
MARTA + tGE8	0.743 (0.614–0.873); P<0.001	0.779 (0.672–0.885); P=0.046	0.540 (0.399–0.682); P=0.62

Panel B. C-index for PFS in IMmotion150

Biomarker	Atezo	Atezo + Beva	Sunitinib
**MARTA**	0.668 (0.591–0.745)	0.628 (0.548–0.707)	0.483 (0.400–0.565)
CXCL13/CXCL8 log-ratio	0.662 (0.585–0.739)	0.618 (0.539–0.697)	0.500 (0.416–0.584)
CXCL13	0.543 (0.463–0.622)	0.584 (0.500–0.669)	0.589 (0.501–0.678)
CXCL8	0.636 (0.553–0.719)	0.593 (0.511–0.674)	0.592 (0.514–0.670)
tGE8	0.543 (0.463–0.623)	0.626 (0.545–0.706)	0.538 (0.441–0.635)
IFN-γ	0.557 (0.474–0.639)	0.623 (0.539–0.707)	0.540 (0.448–0.632)
MARTA + tGE8	0.665 (0.588–0.742); P<0.001	0.649 (0.574–0.723); P=0.094	0.542 (0.443–0.641); P=0.94

Panel C. Discrimination in IMvigor210

Biomarker	ORR AUC (95% CI)	OS C-index (95% CI)
**MARTA**	0.598 (0.519–0.677)	0.572 (0.534–0.611)
CXCL13/CXCL8 log-ratio	0.601 (0.522–0.680)	0.574 (0.535–0.613)
CXCL13	0.602 (0.524–0.679)	0.568 (0.531–0.606)
CXCL8	0.533 (0.454–0.612)	0.530 (0.492–0.568)
tGE8	0.610 (0.532–0.688)	0.546 (0.509–0.584)
IFN-γ	0.613 (0.535–0.691)	0.552 (0.514–0.591)
MARTA + tGE8	0.626 (0.547–0.706); P=0.21	0.572 (0.533–0.610); P=0.001

Values are AUC or Harrell's C-index (95% CI). The alternative non-z-standardised CXCL13/CXCL8 formulation was calculated as log2(TPM_CXCL13 + 1) − log2(TPM_CXCL8 + 1) in IMmotion150 and as the difference between mySimpleVoom-normalised log2 CXCL13 and CXCL8 expression values in IMvigor210. For ORR analyses, higher CXCL8 was prespecified as indicating a lower probability of response; higher values of all other biomarkers indicated a higher probability of response. P values shown for MARTA + tGE8 are from likelihood-ratio tests comparing tGE8 alone with tGE8 + MARTA. Single-gene and signature values were standardised within each cohort. **Abbreviations:** Atezo, atezolizumab; AUC, area under the receiver operating characteristic curve; Beva, bevacizumab; CI, confidence interval; IFN-γ, six-gene interferon-γ signature; MARTA, Myeloid–Adaptive Response to Atezolizumab; ORR, objective response rate; OS, overall survival; PFS, progression-free survival; tGE8, eight-gene T-effector gene-expression signature.

Table S4. Correlations among MARTA and immune-expression biomarkers

Panel A. IMmotion150

Biomarker	MARTA	CXCL13	CXCL8	tGE8	IFN-γ
**MARTA**	1.000	0.496	−0.661	0.424	0.382
CXCL13	0.496	1.000	0.249	0.734	0.715
CXCL8	−0.661	0.249	1.000	0.095	0.141
tGE8	0.424	0.734	0.095	1.000	0.928
IFN-γ	0.382	0.715	0.141	0.928	1.000

Panel B. IMvigor210

Biomarker	MARTA	CXCL13	CXCL8	tGE8	IFN-γ
**MARTA**	1.000	0.639	−0.647	0.442	0.406
CXCL13	0.639	1.000	0.107	0.819	0.779
CXCL8	−0.647	0.107	1.000	0.194	0.213
tGE8	0.442	0.819	0.194	1.000	0.957
IFN-γ	0.406	0.779	0.213	0.957	1.000

Values are Spearman rank correlation coefficients. Analyses included the complete molecular cohorts of 263 patients in IMmotion150 and 347 patients in IMvigor210. **Abbreviations:** IFN-γ, six-gene interferon-γ signature; MARTA, Myeloid–Adaptive Response to Atezolizumab; tGE8, eight-gene T-effector gene-expression signature.

Table S5. Sensitivity analysis according to prior platinum exposure in IMvigor210

Analysis	Deaths, n/N	MARTA HR per 1-SD increase (95% CI)	P value	P for interaction
Overall cohort, adjusted for prior platinum exposure	231/347	0.77 (0.67–0.87)	<0.001	0.71
No prior platinum exposure	41/76	0.81 (0.58–1.12)	0.20	—
Prior platinum exposure	190/271	0.76 (0.66–0.87)	<0.001	—
Multivariate model including prior platinum exposure	172/271	0.81 (0.69–0.95)	0.01	0.10

P for interaction refers to the interaction between continuous MARTA and prior platinum exposure and was obtained by likelihood-ratio comparison of Cox models with and without the interaction term. Prior platinum exposure itself had an HR of 1.34 (95% CI, 0.95–1.88; P=0.09) in the overall model and 1.36 (95% CI, 0.95–1.96; P=0.09) in the multivariate model. The multivariate model was adjusted for tGE8, TMB, PD-L1 immune-cell expression, ECOG PS, and prior platinum exposure. **Abbreviations:** CI, confidence interval; ECOG PS, Eastern Cooperative Oncology Group performance status; HR, hazard ratio; MARTA, Myeloid–Adaptive Response to Atezolizumab; PD-L1, programmed death ligand 1; SD, standard deviation; tGE8, eight-gene T-effector gene-expression signature; TMB, tumour mutational burden.
